# Unique characteristics of acid-tolerant comammox bacteria revealed by multi-omics analyses

**DOI:** 10.1093/ismeco/ycag070

**Published:** 2026-03-23

**Authors:** Tingting Zhang, Junhao Pan, Alejandro Palomo, Zilu Ouyang, Xianghua Wen, Jiyun Li, Chengwen Wang, Min Zheng

**Affiliations:** School of Environment, Tsinghua University, Beijing 100084, China; School of Environment, Tsinghua University, Beijing 100084, China; School of Environmental Science and Engineering, Southern University of Science and Technology (SUSTech), Shenzhen, Guangdong 518055, China; School of Environment, Tsinghua University, Beijing 100084, China; School of Environment, Tsinghua University, Beijing 100084, China; School of Environment, Tsinghua University, Beijing 100084, China; School of Environment, Tsinghua University, Beijing 100084, China; Water Research Centre, School of Civil and Environmental Engineering, University of New South Wales, Sydney, NSW 2052, Australia

**Keywords:** comammox, acid-tolerant, comparative genomics, metatranscriptomics, V-type ATPases

## Abstract

Complete ammonia oxidation (comammox) is a critical biogeochemical process in the nitrogen cycle. In this study, we utilized comammox *Nitrospira* to convert urine wastewater into ammonium nitrate by operating a laboratory-scale membrane bioreactor at pH 3 ~ 4. During the process, the acid-tolerant comammox *Nitrospira* was highly enriched. The metagenomic and metatranscriptomic analyses were applied to reveal its unique characteristics. Comparative genomic analysis among previously reported comammox *Nitrospira* demonstrated that this species was phylogenetically novel, named *Candidatus* Nitrospira aciditolerans. Key mechanisms were further identified to enable this species to thrive in acidic environments. These include active proton efflux, regulation of proton consumption, inhibition of proton influx, and cellular strategies for acid stress management and repair. Remarkably, different from other comammox *Nitrospira* and acid-tolerant ammonia-oxidizing bacteria (AOB), *Candidatus* Nitrospira aciditolerans possesses highly expressed V-type ATPases that are typically associated with acidophilic ammonia-oxidizing archaea (AOA). This may indicate an ecologically significant role for comammox bacteria and AOA in co-maintaining ammonia oxidation activity in low pH environments. Kinetic characterization revealed an apparent ammonium half-saturation coefficient *K*_m_ of 0.50 ± 0.05 *μ*M NH_3_ and an apparent ammonium inhibition constant *K*_i_ of 241.43 ± 45.64 *μ*M NH_3_. The enrichment culture demonstrated optimal ammonia oxidation activity at neutral pH but maintained functionality across a broader pH range between 4 and 8. Like other nitrifying bacteria, this comammox culture was sensitive to temperature and salinity changes. The findings enhance our understanding of the nitrogen cycle under acidic conditions and also present opportunities for engineering applications of acid-tolerant ammonia oxidizers.

## Introduction

Nitrification is a critical biological process in which ammonia is oxidized to nitrate, playing an essential role in both natural environments and engineered ecosystems. Traditionally, this process was understood as a two-step process: ammonia oxidation by ammonia-oxidizing archaea (AOA) or bacteria (AOB), followed by nitrite oxidation by nitrite-oxidizing bacteria (NOB). The discovery of complete ammonia oxidation (comammox), where a single organism can convert ammonia to nitrate, has challenged this conventional view [1–3]. Comammox *Nitrospira* have been detected in various ecosystems, including terrestrial hot springs, soils, mine lake sediments, natural waters, wastewater treatment systems, and drinking water production systems [4–12]. The widespread distribution of comammox bacteria significantly advances our understanding of global nitrogen cycling.

While ammonia-oxidizing organisms typically thrive in slightly alkaline environments [13, 14], certain acidophilic or acid-tolerant ammonia oxidizers have expanded our understanding of nitrification under acidic conditions [15, 16]. Recent studies have detected AOA and comammox *Nitrospira* in acidic environments such as acid mine lake sediments (pH 4.7), where they represent 93% of the total ammonia-oxidizing organisms [5]. Other acidophilic species, such as AOB *Candidatus* Nitrosoglobus terrae, and AOA *Nitrosotalea devanaterra* and *Nitrosotalea sinensis* Nd2, thrive in acidic soils with a pH around 5 [14, 17, 18]. Similarly, several AOB, including *Nitrosomonas*, *Nitrosospira*, and *Nitrosococcus* are capable of growing under acidic conditions in engineered systems, with relative abundances exceeding 2% [19–22].

The ability of ammonia-oxidizing organisms to survive in acidic environments may be attributed to their high affinity for ammonia and specific genomic traits. For example, acid-tolerant mechanisms in AOB involve genes encoding cation transporters, glycosyltransferase, and urea hydrolase [17, 19]. These transporters mediate proton motive forces and regulate pH [19], while glycosyltransferases promote biofilm formation in response to acid stress [23]. In AOA, potassium transporter, Na^+^/H^+^ antiporters, and carbonic anhydrase were postulated to be involved in pH homeostasis, and A-type ATPases and vacuolar type (V-type) ATPases are essential for low pH adaptation in archaea [16, 24]. However, to our knowledge, the mechanisms by which comammox *Nitrospira* adapt to acidic environments remain poorly understood.

Comammox *Nitrospira* have garnered significant attention for their unique nitrification capabilities, yet our understanding of their physiological characteristics remains limited due to the scarcity of pure cultures and enriched cultures [3, 6, 25, 26]. Studies have shown that comammox *Nitrospira* have a high affinity for ammonia, indicating their adaptation to oligotrophic environments [25, 26]. Additionally, comammox *Nitrospira* have distinct affinities for nitrite, with mean apparent half-saturation constants (*K*_m_) for nitrite from 12.5 to 449.2 *μ*M [25, 26]. Environmental factors such as pH, temperature, and salinity also play crucial roles in shaping the distribution of comammox *Nitrospira*, which have been found in environments with pH ranging from 4.7 to 8.4 and temperatures from 8 to 80°C [2, 3, 5, 7, 12].

In this study, we obtained a highly enriched acid-tolerant comammox *Nitrospira* culture. This unique *Nitrospira* culture exhibited ammonia oxidation and nitrification activity under highly acidic conditions (pH 3.1). However, a comprehensive understanding of its genomic and ecophysiological potential was not fully achieved in previous studies [27]. To address this gap, we here employ multi-omics analyses, including genome-centric metagenomic and metatranscriptomic approaches, to further explore unique characteristics of acid-tolerant comammox *Nitrospira*. Additionally, key kinetic parameters, including apparent ammonia affinity and susceptibility to environmental factors such as pH, nitrite, temperature, and salinity, were evaluated through batch tests. Our results reveal ecological significance for comammox bacteria in acidic environments and also have environmental implications for potential biotechnological application.

## Materials and methods

### Bioreactor operation and sampling

A continuous membrane bioreactor (MBR, 16 cm inner diameter, 40 cm height, and 6 L working volume) was established (Fig. S1). The inoculated sludge, sourced from another reactor treating stored urine to enriched comammox *Nitrospira* at pH 6.0 ± 0.1 [27], was used. In Stage I (Day 0 to Day 20), the pH in the reactor was maintained at 6.0 ± 0.1 by an automated pH control system with an online pH meter connected to a dosing pump supplying 1 M NaOH. In Stage II (Day 21 to Day 210), the pH control was removed, allowing natural fluctuations. Additional operational details, including temperature, dissolved oxygen (DO), influent composition, and hydraulic retention time, are provided in Text S1.

Effluent samples were collected every 2–3 days to measure concentrations of NH_4_^+^-N, NO_2_^−^-N, and NO_3_^−^-N. Mixed liquor suspended solids (MLSS) were measured weekly using 25 ml of sludge. Additionally, 15 ml of sludge was centrifuged and stored at −80°C for 16S ribosomal ribonucleic acid (rRNA) gene amplicon sequencing, metagenomic, and metatranscriptomic analyses.

### Deoxyribonucleic acid extraction

DNA was extracted from the eight sludge samples (D0, D18, D46, D73, D113, D145, D173, and D201) using the FastDNA™ SPIN Kit for Soil (MP Biomedicals, CA, USA), following the manufacturer’s protocol. DNA purity was verified via agarose gel electrophoresis and DNA concentration was quantified using a NanoDrop 2000 spectrophotometer (ThermoFisher Scientific, Waltham, MA, USA).

### 16S ribosomal ribonucleic acid gene amplicon sequencing and data analysis

The V3-V4 regions of the microbial 16S rRNA gene sequences from the eight sludge samples were amplified using primers 338F (5′-ACTCCTACGGGAGGCAGCAG-3′) and 806R (5′-GGACTACHVGGGTWTCTAAT-3′). Paired-end sequencing was performed using the Illumina MiSeq PE3000 platform. Raw data were demultiplexed and filtered by QIIME-1.9.1. DADA2 was used for dereplicating the reads. 16S rRNA amplicon sequence variants (ASVs) were taxonomically classified using the SILVA 138/16S database [28]. The abundance tables of the single level (from kingdom to species) were obtained for further analysis of microbial community structure.

### Library construction and metagenomic sequencing

Metagenomic libraries were prepared using the NEXTflex™ Rapid DNA-Seq Kit (Bioo Scientific, Austin, TX, USA) as per the manufacturer’s instructions. Metagenomic sequencing of four bio-samples (D0, D46, D113, and D173) was conducted using NovaSeq Reagent Kits on the Illumina NovaSeq PE150 platform (Illumina Inc., San Diego, CA, USA) at Majorbio Bio-pharm Technology Co., Ltd. (Shanghai, China), generating a total of 80 GB raw data for further bioinformatic analysis.

### Metagenomic assembly and genome binning

High-quality sequencing data were processed using fastp (v0.21.0) and assembled into contigs with SPAdes (v3.15.5) using parameters: -k 33, 55, 77, 99, 127. Contigs (>1.5 kbp) were retained for binning. Metagenomic binning, based on tetranucleotide frequency and sequencing depth, was performed using MetaWRAP (v1.3.2), which integrates MaxBin2 (v2.2.6), MetaBAT2 (v2.12.1), and CONCOCT (v1.0.12) with default settings [29]. The “bin_refinement” module in MetaWRAP was used to refine bin quality, achieving a minimum threshold of 70% completion with a maximum of 10% contamination, as assessed by CheckM (v1.0.12). Six bins were identified as *Nitrospira* using the “classify_wf” workflow of GTDB-Tk (v2.3.2). The relative abundance of genomes was calculated with CoverM (v0.6.1) [30]. The average nucleotide identity (ANI) and amino acid identity (AAI) values were calculated with FastANI (v1.33) and EzAAI tool, respectively.

### Functional annotation and comparative genomic analysis

The functional annotations of high-quality metagenome-assembled genomes (MAGs) were conducted using Prodigal (v2.6.3) in conjunction with GhostKOALA (v2.0) and eggNOG-mapper (v2.1.6). Comparative genomic analysis was carried out with OrthoFinder (v2.5.5), comparing specific genomic features related to acid stress between the recovered comammox genomes and published genomes of ammonia-oxidizing organisms, including comammox *Nitrospira*, acidophilic/acid-tolerant AOA, and AOB.

### Phylogenetic analysis

Phylogenetic analyses were performed on the 16S rRNA gene tree, *amoA* gene, and recovered comammox genomes, with trees constructed accordingly.

#### 16S ribosomal ribonucleic acid gene tree

Phylogenetic analysis was performed on nitrifier ASVs (*Nitrospira*) from the bioreactor. Reference sequences of *Nitrospira* were downloaded from NCBI-RefSeq database. Sequence alignments were generated using MSCLE (v5.1) [31] with default parameters. Maximum likelihood (ML) tree construction was performed using IQ-TREE (v2.2.2.3) [32], with the TIM3 + F + R3 model selected via automatic model search (−nt AUTO) and ultrafast bootstrapping with 1000 replications (−bb 1000). The final tree was visualized using iTOL v6 (https://itol.embl.de/).

#### AmoA gene tree

An internal database of 42 *amoA* protein sequences, representing AOB, AOA, and comammox *Nitrospira*, was constructed from NCBI. These sequences were used to align with *amoA* protein sequences from the assembled contigs and MAGs via BLASTx (v2.14.0). Alignments exceeding 200 amino acids in length and showing >90% identity were extracted for phylogenetic analysis. The ML tree of the *amoA* gene was constructed using MSCLE (v5.1) for alignment and IQ-TREE (v2.2.2.3) for tree generation, applying the LG + R2 model determined through automatic model selection (−nt AUTO) and 1000 ultrafast bootstrap replications (−bb 1000). The resulting tree was visualized via iTOL v6. The relative abundance of the *amoA* genes was calculated using CoverM (v0.6.1).

#### Genome tree

To resolve sublineage-level phylogeny of high-quality MAGs derived from metagenomic binning, 58 common *Nitrospira* MAGs were downloaded from the NCBI database. Using *Candidatus* Nitronauta litoralis as the outgroup, 120 single-copy marker genes (MGs) from the 63 MAGs were aligned using GTDB-Tk (v2.3.2) under default settings. The best-fit phylogenetic model (F + I + R5) was determined using the ModelFinder module of IQ-TREE (v2.2.2.3). The final phylogenomic tree was constructed with 1000 bootstrap iterations and visualized using iTOL v6.

### Metatranscriptomic analysis

To explore the gene expression of comammox *Nitrospira* under extremely acidic condition, total RNA of two samples from different culturing stages (one sample from Day 10 in Stage I (pH 6 ~ 7) and one sample from Day 139 in Stage II (pH 3 ~ 4), in technical triplicate) was extracted using the Soil RNA Extraction Kit (Majorbio, shanghai, China) and metatranscriptomic sequencing was conducted on a DNBSEQ-T7 platform at Majorbio Bio-pharm Technology Co., Ltd. (Shanghai, China). A total of 120 Gbps of raw sequence data were preprocessed using fastp (v0.23.4), and ribosomal RNA reads were removed using SortMeRNA (v4.3.6). The remaining mRNA reads were then competitively mapped to previously assembled four comammox *Nitrospira* MAGs by bowtie2 (v2.5.1). Considering the differentiated gene lengths and sequencing depths, gene expression levels in transcripts per kilobase per million mapped reads (TPM) were calculated using CoverM (v0.6.1). During comparative analysis between pH 3 ~ 4 and 6 ~ 7, TPM was normalized to relative cell numbers by dividing the transcript abundances by the median abundance of 10 universal single-copy phylogenetic MGs. Differential gene expression analysis was conducted with DESeq2. Differential gene expression >2-fold expression and *P*-values <0.05 were regarded as significant.

### Batch experiments

Between Days 155 and 195 of reactor operation, batch tests were conducted to evaluate the apparent total ammonium affinity and the effects of various environmental factors (pH, FNA, temperature, and salinity) on the ammonia oxidation activity of the enriched culture. The conditions for each batch test are provided in Table S1. 15 ml of sludge were collected from the reactor, centrifuged for 10 min at 5000 rpm, and washed three times with 1X PBS solution to remove residual nitrogen compounds. Each batch test was carried out in a 250 ml conical flask sealed with breathable film, shaken at 150 rpm under controlled temperature conditions using a thermostatic oscillator. The pH was adjusted using 1 M NaOH and HCl solutions, while NH_4_Cl, NaNO_2,_ and NaCl were added to achieve desired initial concentrations of NH_4_^+^-N, NO_2_^−^-N, and salinity, respectively. Additionally, 0.2 ml each of trace element (Table S2) and 0.14 M NaHCO_3_ solution were added to the system, with the total volume brought to 100 ml using 1X PBS. All tests were conducted in duplicate.

The incubation time for every batch test was 48 hours. Samples of mixed liquor were taken every 12 h for a total of 5 times, filtered through a 0.45 *μ*M aqueous phase needle filter (ANPEL, Shanghai), and analyzed for NH_4_^+^-N, NO_2_^−^-N and NO_3_^−^-N concentrations. The pH of the reaction system was monitored and adjusted as necessary. The specific reaction rate was used to assess the ammonia oxidation activity of comammox *Nitrospira*. The calculations for the mean apparent inhibition constant (*K*_i_) and mean apparent half-saturation constant (*K*_m_) are detailed in Text S2.

### Chemical analysis and calculation

Concentrations of NH_4_^+^-N, NO_2_^−^-N, NO_3_^−^-N, MLSS, and mixed liquor volatile suspended solids (MLVSS) were measured using standard methods [33]. pH was measured using a DG150 pH meter (Lohand Biological, Hangzhou, China), and DO and temperature were recorded with a multi-parameter meter (WTW Multi 3420i, Germany). Free ammonia (FA) and FNA concentrations were calculated using established methods [34].

## Results and discussion

### Cultivation of comammox *Nitrospira* in low potential-of-hydrogen environment

After sludge inoculation, the influent ammonium (590 ± 34 mg N/L) was completely converted to nitrate with minimal nitrite accumulation (<0.2 mg N/L) during the transitory pH-control stage (Fig. S2A). Once pH control was ceased, the pH naturally decreased and stabilized between 3 and 4 (Fig. S2B). Meanwhile, around half of the ammonium was converted to nitrate, realizing the successful production of ammonium nitrate from urine wastewater. The calculated free ammonia concentrations were consistently below 0.05 *μ*M (Fig. S2B). Previous research has demonstrated that comammox *Nitrospira* possess a higher ammonia affinity compared to most characterized AOB and many non-marine AOA [14, 25, 26]. Thus, the oligotrophic environment, with low FA concentration, might favor the dominance of comammox *Nitrospira* over canonical AOB and NOB.

To assess the microbial community and confirm the dominance of comammox *Nitrospira* in the MBR, 16S rRNA gene amplicon sequencing and phylogenetic analysis were conducted. As shown in Fig. S3, *Proteobacteria* dominated the microbial community, with *Nitrospira* comprising ~20% of the community at pH 3 ~ 4. Notably, canonical AOB genera (e.g. *Nitrosomonas*, *Nitrosospira*, and *Nitrosoglobus*) and common NOB genera (e.g. *Nitrobacter* and *Nitrotoga*) were absent, indicating that *Nitrospira* were likely the sole nitrifiers. Phylogenetic analysis of 16S rRNA gene sequences further suggested that three ASVs (ASV1, ASV383, and ASV791) belonged to *Nitrospira* sublineage II and were closely related to other comammox clade A sequences, suggesting that these ASVs likely represented comammox *Nitrospira* (Fig. S4). Meanwhile, two ASVs (ASV386 and ASV399) with very low abundance were closely phylogenetically related to canonical NOB *Nitrospira defluvii* in *Nitrospira* sublineage I (Fig. S4).

The dominance of comammox *Nitrospira* was corroborated by *amoA* gene sequence analysis from metagenomic data, as 16S rRNA gene amplicon sequencing alone cannot definitively distinguish comammox *Nitrospira* from canonical NOB *Nitrospira*. Five *amoA* sequences were retrieved from assembled contigs, aligned with an internal database, all showing >90% identity. Four of these *amoA* sequences clustered phylogenetically with known comammox *Nitrospira* clade A, and their combined relative abundance accounted for >99.8% of all detected *amoA* sequences (Fig. S5). Only one *amoA* sequence, which has very low abundance, was affiliated with the AOB *Nitrosospira* genus. These results confirmed that comammox *Nitrospira* were the predominant ammonia oxidizers in the system, particularly under low-pH conditions of 3 ~ 4.

### Metagenomic retrieval of comammox *Nitrospira*

Metagenomic sequencing, followed by assembly and binning, resulted in the recovery of 73 high-quality MAGs (completeness ≥90% and contamination <5%) and 24 medium-quality MAGs (completeness ≥70% and contamination <5%) (Dataset S1). Among these, three high-quality MAGs (BIN78, BIN85, BIN64) and one medium-quality MAG (BIN8) were affiliated with comammox *Nitrospira*. These MAGs contained the essential functional genes required for complete nitrification, including *amoCAB* (ammonia monooxygenase), *hao* (hydroxylamine dehydrogenase), and *nxrAB* (nitrite oxidoreductase). Additionally, two high-quality MAGs were identified as belonging to canonical NOB *Nitrospira*. However, the relative abundance of NOB *Nitrospira* was three orders of magnitude lower than that of comammox *Nitrospira*, making their impact negligible (Table 1). Consistent with the results of 16S rRNA gene sequencing, no AOA or AOB MAGs were recovered from the reactor. The genomic information of the four comammox *Nitrospira* MAGs is summarized in Table 1.

**Table 1:** Genomic information of four comammox *Nitrospira* MAGs constructed in this work.

Bins		Completeness (%)	Contamination (%)	GC content (%)	Genome size (bp)	Number of contigs	Largest contig size (bp)	N50 value (bp)	Relative abundance			
									D0	D46	D113	D173
BIN78	Comammox	95.8	3.2	55.7	4 566 574	160	194 165	70 783	25.08	26.89	22.40	39.04
BIN85		94.9	1.8	56.0	4 519 799	90	293 498	93 891	14.12	2.14	0.59	1.51
BIN64		93.2	4.5	55.4	4 511 734	124	210 229	57 867	0.77	0.31	0.18	0.66
BIN8		86.2	2.4	56.0	4 196 467	351	81 243	17 827	1.35	0.41	0.21	0.45
BIN57	NOB	96.8	3.6	58.7	4 497 480	97	411 496	126 525	0.47	0.90	0.00	0.35
BIN82		96.8	2.7	59.5	4 084 529	35	384 641	242 224	0.32	0.24	0.00	0.04

**Figure 1:**
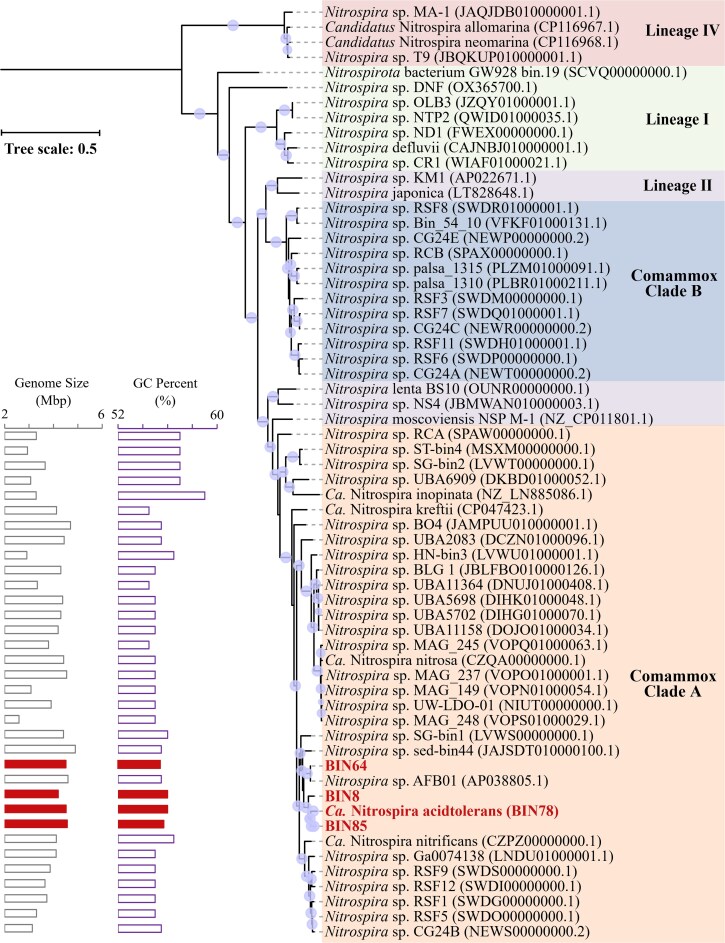
Phylogenomic tree of the Ca. Nitrospira aciditolerans MAG obtained in this study, with *Nitronauta* as the outgroup. Purple circles indicate bootstrap values ≥70%. The GenBank accession number of the corresponding *Nitrospira* is noted in parentheses. Genome size and GC percent of each comammox clade A *Nitrospira* genome are shown on the left side of the phylogenomic tree.

**Figure 2:**
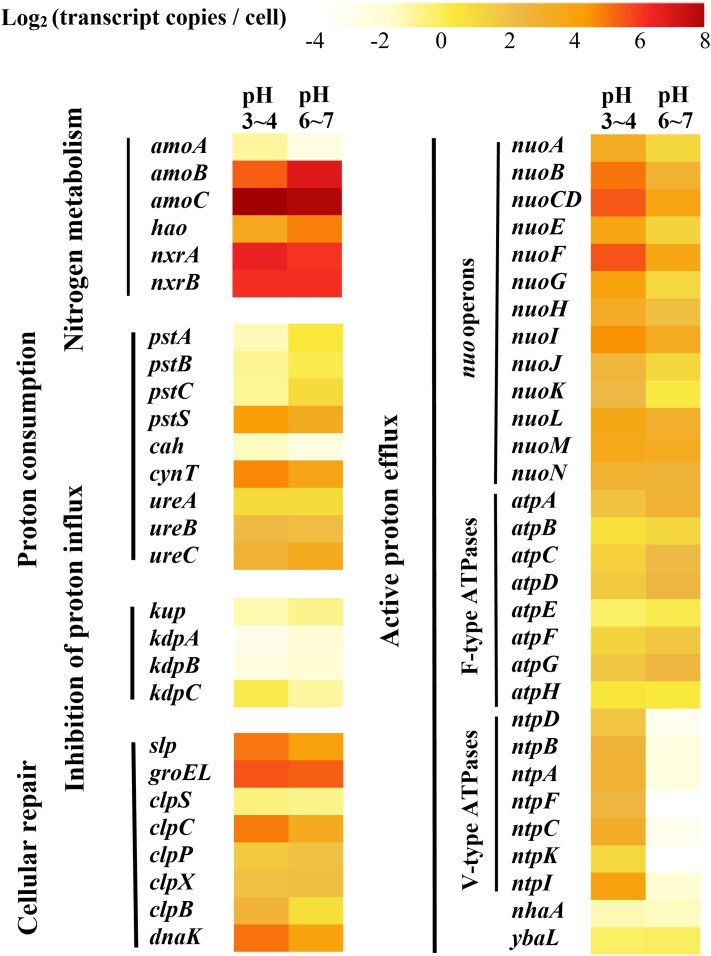
Differential gene expression profiles of Ca. Nitrospira aciditolerans involved in nitrogen metabolism and acid adaptation. The heatmap showed the differences in the mean transcript abundances per organism under acidic (pH 3 ~ 4, left) versus near-neutral (pH 6 ~ 7, right) conditions. Differences were computed using log2- transformed values.

Phylogenomic analysis revealed that all four comammox *Nitrospira* MAGs belonged to clade A of the comammox *Nitrospira* group (Fig. 1). Their combined relative abundance remained almost unchanged (from 41.3 to 41.7%) during reactor operation. Notably, one specific comammox *Nitrospira* MAG (BIN78) was predominant during the entire culture period, even reaching a relative abundance of 39.0% by Day 173 of reactor operation (Table 1). Generally, neutral environments are beneficial for ammonia oxidizers including comammox *Nitrospira*. However, the inoculated sludge and the initial conditions (Stage I, Day 0 to Day 20) both had a lower pH (6.0) than the optimal conditions for other ammonia oxidizers (~7.5). This might explain the enrichment of comammox *Nitrospira* MAG (BIN78) in the Stage I. And the *amoA* gene sequence of BIN78 is identical to *amoA* 1# gene sequence from assembled contigs. Meanwhile, compared with other comammox *Nitrospira* MAGs, higher transcription levels of *amoABC*, *hao*, and *nxrAB* were observed in BIN78, whether in acidic or nearly neutral environments (Fig. 2). Both the ANI and AAI values between BIN78 and publicly available genomes were below the established species threshold of 95% (Fig. S6) [35, 36], indicating that the MAG represents a novel comammox *Nitrospira* species. Based on its unique genetic composition and acid tolerance, we propose the tentative name *Candidatus* (Ca.) Nitrospira aciditolerans for this novel organism.

### Genomic adaptations of *Candidatus* Nitrospira aciditolerans to acidic environments

Ammonia oxidation typically occurs at pH levels above 5.5, with few exceptions such as comammox *Nitrospira* found in acid lake sediments, the acid-tolerant bacteria *Candidatus* Nitrosoglobus, *Candidatus* Nitrosacidococcus*,* and the archaeon *Candidatus* Nitrosotalea, which thrive at pH levels below 5 [5, 16, 19, 37]. In this study, the enriched Ca. Nitrospira aciditolerans demonstrated the ability to oxidize ammonium at pH levels as low as 3.1. Comparative genomic and transcriptional analyses were used to seek to uncover its unique genomic characteristics.

In bacteria, pH homeostasis is often regulated by the proton motive force (PMF), which is generated through the combination of the transmembrane pH gradient (ΔpH) and the transmembrane electrical potential (Δψ). Acidophilic and acid-tolerant microorganisms typically maintain a higher ΔpH (i.e. an internal pH that is greater than the external pH), partially counterbalanced by a reverse Δψ (where the inside of the cell is more positive relative to the outside) to maintain stability [23]. Several key strategies are possibly employed by these microorganisms to maintain a higher internal pH (generally above 6), including active proton efflux, regulation of proton consumption, inhibition of proton influx, various cellular stress and repair mechanisms to cope with acid stress.

#### Coping with low potential-of-hydrogen challenges through direct active proton efflux

Transporters play a critical role in the active efflux of protons, which helps cells manage acidic environments. These include primary proton pumps and secondary active transporters. Primary proton pumps, such as respiratory chain pumps or proton-pumping ATPases, help generate and maintain PMF in bacteria, while secondary transporters like cation-proton antiporters facilitate the exchange of protons for other cations across the membrane. Interestingly, compared with other *Nitrospira* that only contain two or three sets of *nuo* operons [38], Ca. Nitrospira aciditolerans encodes four complete homologous sets of the *nuo* operons, which encode complex I in the electron transport chain. Transcriptional profiling revealed that Ca. Nitrospira aciditolerans exhibited higher transcriptional levels of the *nuo* operons under acidic conditions compared to near-neutral pH conditions (Fig. 2). This complex is known to couple proton pumping to electron transfer, enabling cells to expel protons in acidic conditions [23]. Formic acid (released by other organisms) or H_2_ could be potential electron donors for the process. This is supported by the presence and upregulation of key metabolic genes in the Ca. Nitrospira aciditolerans that are potentially associated with these processes, e.g. *fdhA* for formic acid catabolism, and the hydrogenase (encoded by *hyfBCEF*) that might facilitate H_2_ oxidation (Fig. S7).

**Figure 4:**
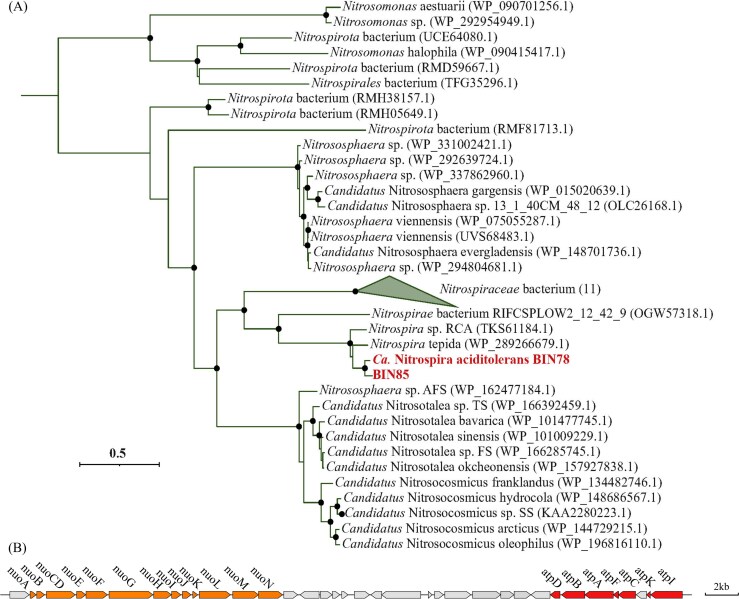
Classification and phylogeny of V-type ATPase harbored by the Ca. Nitrospira aciditolerans genome. (A) the maximum likelihood tree shows the phylogenetic affiliation of the subunit A of V-type ATPase encoded by Ca. Nitrospira aciditolerans genome and reference sequences. Bootstrap values (based on 1000 iterations) ≥ 80% are indicated by black circles. (B) Schematic illustration of the genomic loci of the V-type ATPase with genes *atpA-I* of Ca. Nitrospira aciditolerans genome. Genes in orange states for complex I and in red for encoding V-type ATPase. Genes are drawn to scale.

**Figure 3:**
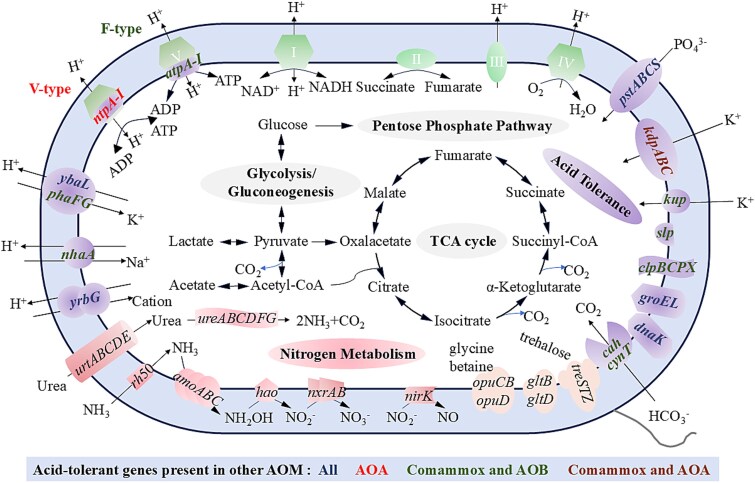
Cell metabolic diagram constructed from the Ca. Nitrospira aciditolerans genome enriched in this work. Only genes relevant to carbon, nitrogen, energy metabolisms, and acid tolerance are exhibited. Solid lines indicate genes detected in genome of Ca. Nitrospira aciditolerans retrieved in this study.

With regard to ATPases, proton-pumping ATPase utilize ATP hydrolysis to drive proton extrusion under acidic conditions [39]. Notably, the Ca. Nitrospira aciditolerans genome encodes both F-type and V-type ATPases (Fig. 3). To date, only a small percentage (4.8%) of *Nitrospirales* encode a V-type ATPase, including NOB *Nitrospira* (*Nitrospira* tepida, Ca. Nitrospira bockiana, Ca. *Nomia calida*, and *Nitrospira* sp. Kam-Ns4a) and one known comammox *Nitrospira* species (*Nitrospira* sp. RCA) [40]. NOB *Nitrospira* were widespread in environments with elevated temperatures, and *Nitrospira* sp. RCA originated from the sediment of the Rifle site (a former uranium-rich milling facility adjacent to the Colorado River) [41–44]. V-type ATPases resembling A-type ATPases are found in archaeal acidophiles, which are prevalent in all acidophilic AOA (Fig. 3). A recent study demonstrated that the expression of V-type ATPases correlates with increased proton pumping activity in acid-tolerant *thaumarchaeotea* [16]. In this work, Ca. Nitrospira aciditolerans displayed higher levels of V-type ATPases expression under acidic conditions (Fig. 2), indicating their important roles in acidic adaptation. Phylogenetic analyses (Figs 4A, S8) revealed that all subunits of V-type ATPase encoded by Ca. Nitrospira aciditolerans were closely related to those in acid-tolerant AOA, such as *Candidatus* Nitrosocosmicus oleophilus, *Nitrososphaera* sp. AFS, and *Candidatus* Nitrosotalea sp. TS. The arrangement of V-type ATPase subunits in Ca. Nitrospira aciditolerans is similar to Ca. Nitrosocosmicus oleophilus and *Nitrosotalea devanaterra* (Fig. S9). This suggests that Ca. Nitrospira aciditolerans can export excess protons in low pH environments, much like other acid-tolerant AOA. Interestingly, the *atpA-I* genes encoding V-type ATPases are located in the same genomic region as the fourth complete *nuo* operon in Ca. Nitrospira aciditolerans (Fig. 4B). These genomic characteristics were also identified in the closely related comammox MAG BIN85. Transcriptional profiling also revealed the higher gene expression level of BIN85 under acidic conditions (Fig. S10). The comammox MAG BIN85 and Ca. Nitrospira aciditolerans (BIN78) had similar gene repertory, however, the relative abundances (<2%) of BIN85 dropped sharply under acidic conditions (Table 1). Notably, Ca. Nitrospira aciditolerans (BIN78) was already more abundant than BIN85 at Day 0 (Table 1). The precipitous decrease of BIN85 under acidic conditions (Stage II) might be due to the fact that early colonizers (Ca. Nitrospira aciditolerans) shaped environmental niches, influencing the success of later competitors [45]. In addition, the presence of these genes in the other two comammox MAGs, BIN64 and BIN8, each with an abundance of <1%, remains uncertain. Their lower genome completeness (93% for BIN64 and 86% for BIN8) may partly explain the apparent absence of these genes. Alternatively, the greater genetic divergence of these MAGs from Ca. Nitrospira aciditolerans (BIN78) suggests that species-level differences could account for the genuine absence of these genes. These findings suggest that V-type ATPases could be crucial for maintaining ammonia oxidation activity under acidic conditions.

The Ca. Nitrospira aciditolerans genome also encodes several monovalent cation-dependent transporters, including secondary Na^+^/H^+^ antiporters (encoded by gene *nhaA*) and K^+^/H^+^ antiporters (encoded by *ybaL*), both of which play a potential role in pH homeostasis [23]. Na^+^(K^+^)/H^+^ antiporters are common among both extremophilic and non-extremophilic organisms [46]. While these antiporters have been traditionally associated with pH regulation in alkaline environments [47], transcriptional data showed that in acidic conditions, the Ca. Nitrospira aciditolerans could also use Na^+^(K^+^)/H^+^ antiporters to pump out excess protons (Fig. 2). Similar mechanism was also reported by Hua *et al.* [48], suggesting that these transporters are vital mechanisms for maintaining pH balance in acidic environments.

#### Enhancing intracellular potential of hydrogen through proton consumption

Another key strategy for increasing intracellular pH involves proton consumption through metabolic adjustments [23]. Phosphate, a common proton-buffering molecule, can be transported into the cell by the phosphate transport system (*pstSCAB*) [49–51]. The genes encoding this system are conserved across all ammonia-oxidizing microorganisms, including Ca. Nitrospira aciditolerans genome. Differential gene expression analysis indicated that the expression of *pstSCAB* genes in the Ca. Nitrospira aciditolerans genome was upregulated in acidic conditions, supporting its possible role in acid stress mitigation (Dataset S2). Besides, carbonic anhydrase (*cah* and *cynT*) facilitates the conversion of CO_2_ to HCO_3_^−^, while urease (*ureABC*) promotes the breakdown of urea from supplied stored urine into CO_2_ and NH_3_. In Ca. Nitrospira aciditolerans, the combined expression of these enzymes could generate ammonium bicarbonate, which acts as a periplasmic buffer—a mechanism analogous to that observed in *Helicobacter pylori* [52].

#### Maintaining potential of hydrogen homeostasis by repelling proton influx

Limiting proton influx is also a key strategy to maintain pH homeostasis. Generally, acidophiles can adapt to acidic environments by generating a positive internal potential that prevents proton inflow [53]. The Ca. Nitrospira aciditolerans genome encodes both potassium uptake proteins (*kup*) and a potassium-transporting ATPase system (*kdpABC*) (Fig. 3). Comparative genomic analysis indicated that these potassium transporters are also present in other neutrophilic comammox *Nitrospira* (Fig. 3). This suggests that potassium homeostasis might be a fundamental physiological trait in the comammox *Nitrospira*. For Ca. Nitrospira aciditolerans, it is more plausible that the uptake of K^+^ repels proton influx and prevents cytoplasmic acidification, rather than fundamentally reversing membrane potential. Transcriptional upregulation of these genes confirmed that these mechanisms contribute to its adaptation to acidic environments (Dataset S2).

#### Addressing acid shock through cell protection and repair

In addition to regulating protons, certain repair mechanisms help cells cope with acid shock. Low pH can damage cell membranes, leading to changes in cell morphology [54]. The gene *slp* encodes an outer membrane lipoprotein that forms a crystalline protein layer, stabilizing cell morphology and enhancing acid tolerance by maintaining membrane integrity and fluidity [55, 56]. Furthermore, chaperone proteins such as *groEL*, *clpBCP*, and *dnaK* assist in protein repair during stress conditions [57, 58]. These repair-related genes were present in the Ca. Nitrospira aciditolerans genome and highly expressed (Figs 2 and 3), suggesting that cell protection and repair are important mechanisms for the novel comammox *Nitrospira* species to cope with acid stress.

Additionally, apart from Ca. Nitrospira aciditolerans, the microbial community also contained abundant *Mycobacterium* and *Rhodanobacter* under acidic conditions, with a relative abundance reaching up to 43.48% and 33.93%, respectively. They could also play the potential roles for the acid adaptation of Ca. Nitrospira aciditolerans. For example, transcriptional data showed that the genes related to oxidative stress in *Mycobacterium* were activated and expressed at a high level under acidic conditions, such as *oxyR*, *katG*, and *ahpC* (Fig. S11). *OxyR*, the central regulator of the bacterial oxidative stress response, regulates the gene *katG* encoding the enzyme with catalase-peroxidase function and *ahpC* encoding alkyl hydroperoxide reductase in response to peroxide-induced stress [59, 60]. So, *Mycobacterium* could support Ca. Nitrospira aciditolerans in this acidic system by protecting them from oxidative stress. Another possible reason was that heterotrophic bacteria *Rhodanobacter* secreted extracellular polymers (EPS) and were helpful in the formation of biofilms and cell aggregates [61]. This physical barrier could help Ca. Nitrospira aciditolerans maintain activity at low pH.

### Characterization of the *Candidatus* Nitrospira aciditolerans enrichment culture

**Figure 5:**
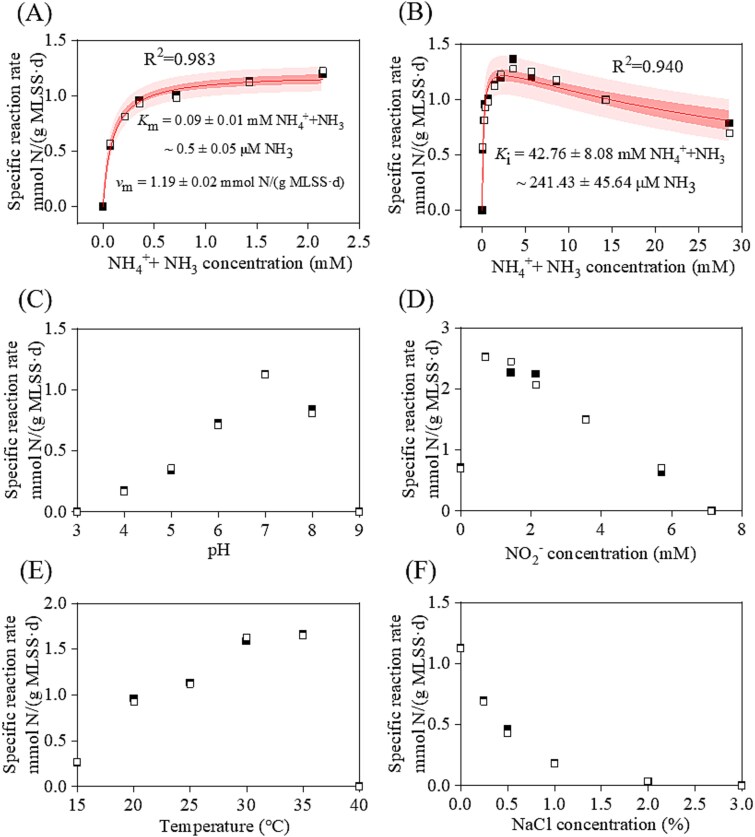
The apparent ammonium affinity and effects of environmental factors on the ammonia oxidation activity of the Ca. Nitrospira aciditolerans enrichment culture. The red lines and shaded areas show the best fit of (A) the data retrieved for non-inhibitory ammonium concentrations based on a Michaelis–Menten kinetic equation and (B) all data to the substrate inhibition model and the resultant 95% confidence bands, respectively. The effects of pH (C), NO_2_^−^-N concentration (D), temperature (E), and salinity (F) on the activity of the enriched culture are presented, respectively. Results of two biological replicates are shown by the solid boxes and the hollow boxes, respectively.

Although the obtained Ca. Nitrospira aciditolerans is not a pure culture and has certain limitations for characterization, its apparent kinetic parameters are of great significance for our understanding of the performance of the enrichment culture under acidic conditions. Batch activity tests were conducted to assess its apparent affinity to ammonium and its response to environmental factors. Using Michaelis–Menten kinetics, the mean apparent half-saturation constant (*K*_m_) was determined to be 0.50 ± 0.05 *μ*M NH_3_ (equivalent to ~0.09 ± 0.01 mM NH_4_^+^+NH_3_), while the mean maximum total ammonium oxidation rate (*ν*_m_) was 1.19 ± 0.02 mmol N/(g MLSS·d) at pH 7 (Fig. 5A). The results indicated that the Ca. Nitrospira aciditolerans enrichment culture had a lower ammonia affinity than other known comammox *Nitrospira* (Table S3). This might be due to cellular effects of the long-term acidic environment [14]. Moreover, when the ammonium concentration exceeded 2.14 mM, an apparent inhibition constant (*K*_i_) of 241.43 ± 45.64 *μ*M NH_3_ (corresponding to 42.76 ± 8.08 mM NH_4_^+^+NH_3_) was observed (Fig. 5B). This suggests that the Ca. Nitrospira aciditolerans enrichment culture exhibits higher adaptation to high-ammonium environments compared to other comammox species, including Ca. Nitrospira kreftii and *Nitrospira* sp. B04 [26, 62].

Similar to most ammonia-oxidizing microorganisms (Table S3), the Ca. Nitrospira aciditolerans enrichment culture exhibited optimal ammonia oxidation activity at pH 7 (Fig. 5C), achieving a specific reaction rate of 1.12 mmol N/(g MLSS·d). Remarkably, the culture maintained the ammonia oxidation activity even under acidic conditions, with activity persisting down to pH 4 (Fig. 5C), consistent with that the culture was enriched in the low-pH bioreactor. Only a few AOB, such as Ca. Nitrosacidococcus tergens and Ca. Nitrosoglobus terrae, have been shown to survive at pH levels below 3 [17, 19]. Under alkaline conditions, the ammonia oxidation rate gradually decreased as pH increased, but activity did not cease until the pH reached 9, indicating that the enrichment culture can adapt to a broad pH range.

Previous studies have demonstrated that FNA can inhibit or inactivate nitrifying microorganisms [37, 63, 64]. This study confirmed that elevated nitrite concentration had an inhibitory effect on the Ca. Nitrospira aciditolerans enrichment culture. The ammonia oxidation activity decreased linearly as the nitrite concentration increased from 0.71 to 7.14 mM, corresponding to the FNA concentration of 1.43 to 15.71 *μ*M (Fig. 5D). Compared to the Ca. Nitrosoglobus enrichment culture, which can tolerate up to 5.09 mM HNO_2_-N [37], the Ca. Nitrospira aciditolerans enrichment culture exhibited a more limited FNA tolerance.

Previous research has shown that comammox *Nitrospira* species are highly adaptable to a wide range of temperatures, from 8 to 80°C [65]. The Ca. Nitrospira aciditolerans enrichment culture exhibited peak ammonia oxidation activity at 35°C but became inactive at 40°C (Fig. 5E). According to the genome draft, Ca. Nitrospira aciditolerans possesses genetic capacity for the synthesis and transport of osmoprotectants like trehalose (*treS*, *treT*, and *treZ*), glycine betaine (*opuABCD*), and glutamate (*gltB* and *gltD*). However, the Ca. Nitrospira aciditolerans enrichment culture still showed a high sensitivity to salinity, losing 60% of its activity when exposed to 0.5% NaCl (Fig. 5F), in contrast to AOB genera such as *Nitrosococcus*, *Nitrosomonas*, *Nitrosospira*, and *Nitrosoglobus*, which maintain activity at 2% NaCl [37, 66–68]. This salinity sensitivity is similar to that of comammox *Nitrospira inopinata*, which ceased to grow at 1.28% NaCl during ammonia oxidation [69].

### Ecological and environmental implications

The study reported the first genomic characterization of acid-tolerant comammox bacteria. Consistent with other ammonia oxidizers in acidic environments, cation transporters (Na^+^/H^+^, K^+^/H^+^, etc.) play a potential role in pH homeostasis [16, 17]. The presence of carbonic anhydrase and urease is involved in proton scavenging [17, 19]. According to comparative genomics, the genomes of acid-tolerant ammonia oxidizers, including Ca. Nitrospira aciditolerans, all harbored the potassium uptake proteins or potassium-transporting ATPase system, maintaining pH homeostasis by repelling H^+^ influx.

However, different from other comammox *Nitrospira* and acid-tolerant AOB, Ca. Nitrospira aciditolerans genome contains highly expressed V-type ATPases, which are typically associated with acid adaptation and are generally found in acidophilic AOA but rarely observed in known comammox *Nitrospira* species. Furthermore, compared to other *Nitrospira*, the Ca. Nitrospira aciditolerans genome has one extra *nuo* operon encoding the NADH dehydrogenase genes (*nuoA-N*), and the *atpA-I* genes of the V-type ATPases are exactly located in the genomic region containing the complete *nuo* operon. The co-localization of the genes for the *nuo* operon and the V-type ATPase indicated that this gene cluster may be controlled by the same promoter or be under the same regulatory network. When cells sense acidic stress, they can simultaneously synthesize the *nuo* operon and the V-type ATPase, achieving the optimal physiological response efficiency. This may indicate an ecologically significant role for comammox bacteria and AOA in co-maintaining ammonia oxidation activity in low pH environments.

Further, we checked that only the other comammox MAG available, *Nitrospira* sp. RCA of the comammox clade A, harbored the genes encoding V-type ATPases [41]. Indeed, *Nitrospira* sp. RCA was also obtained from a harsh environment, i.e. the sediment of a former uranium-rich milling facility [41]. This means that V-type ATPases, likely obtained through horizontal transfer, could be essential for these comammox bacteria to cope with complex environments. In a previous report, Li *et al.* indeed found that comammox *Nitrospira* and AOA were dominant ammonia oxidizers in the acid mine lake sediments (pH 4.7) [5]. Comammox *Nitrospira* species were also found in the acidic biofilm reactor (pH 5) and enriched from acidic soil at pH <5.5 [70, 71]. Collectively, the discovery of Ca. Nitrospira aciditolerans has not only expanded microbial diversity in extreme ecosystems but also deepened our understanding of microbial adaptation and ecological resilience under environmental stress. And the discovery also presents opportunities for engineering applications of acid-tolerant ammonia oxidizers, such as the recovery of ammonium nitrate.

## Supplementary Material

SI_0319_ycag070

Dataset_S1_ycag070

Dataset_S2_ycag070

## Data Availability

16S rRNA gene amplicon sequencing, metagenomic sequencing data, the assembly, the obtained MAGs, and the transcriptome data are all available at the NCBI under project PRJNA1132894 (https://www.ncbi.nlm.nih.gov/bioproject/PRJNA1132894).
