# Supplementary material for: Unique characteristics of acid-tolerant comammox bacteria revealed by multi-omics analyses
Source: ISME Commun. 2026 Mar 23;6(1):ycag070. doi: 10.1093/ismeco/ycag070 (PMC13109103; doi:10.1093/ismeco/ycag070)
Supplement: SI_0319_ycag070 [file si_0319_ycag070.docx]

**Supporting information**

**Unique characteristics of acid-tolerant comammox bacteria**

**revealed by multi-omics analyses**

Tingting Zhang^1^, Junhao Pan^1^, Alejandro Palomo^2^, Zilu Ouyang^1^, Xianghua Wen^1^, Jiyun Li^1,*^, Chengwen Wang^1,*^, and Min Zheng^3,*^

^1^ School of Environment, Tsinghua University, Beijing 100084, China

^2^School of Environmental Science and Engineering, Southern University of Science and Technology (SUSTech), Shenzhen, Guangdong 518055, China

^3^Water Research Centre, School of Civil and Environmental Engineering, University of New South Wales, Sydney, New South Wales 2052, Australia

* **Corresponding authors**:

Email addresses: [lijiyunn@163.com](mailto:lijiyunn@163.com) (Jiyun Li); [wangcw@tsinghua.edu.cn](mailto:wangcw@tsinghua.edu.cn) (Chengwen Wang); [min.zheng1@unsw.edu.au](mailto:min.zheng1@unsw.edu.au) (Min Zheng)

Number of pages: 17

Number of tables: 3

Number of figures: 11

**Text S1.** **Bioreactor operation and sampling**

The reactor was made of plexiglass and installed with a hollow fiber ultrafiltration membrane module (Originwater, Beijing, China), whose pore size and total surface area were 0.01 *μ*m and 0.02 m^2^, respectively. The whole culture process was carried out at room temperature. Sufficient oxygen was constantly provided by an aeration pump at a rate of 1.5 L/min. The influent and effluent pumps were regulated by an automatic control system [1]. The real stored urine wastewater was used for the feed, whose total nitrogen concentration was around 600 mg N/L (616±22 mg N/L). Nitrogen in stored urine wastewater mainly existed in the form of ammonium while the urea concentration was about 40 mg N/L. There was no nitrite in the influent and the nitrate concentration was below 10 mg N/L. With a daily effluent volume of 1 L, the hydraulic retention time (HRT) was 6 days. No sludge was discharged except for sampling and testing during the whole operation.

**Text S2. Batch experiments**

The ratio of NH_4_^+^-N degradation rate or NO_3_^-^-N production rate to MLSS concentration, which is the specific reaction rate, was used to characterize the activity of comammox *Nitrospira*. The relationship between the specific reaction rate and the substrate (NH_4_^+^-N) concentration was best described by the Haldane substrate inhibition model (Eq. (1)) and the *K*_i_ value was calculated based on fitting the data to this model. Meanwhile, by fitting the experimental data obtained for non-inhibitory ammonium concentrations to a Michaelis-Menten model (Eq. (2)), the *K*_m_ and *v*_m_ values were calculated.

$v=\frac{v_{\text{m}}S}{K_{\text{m}}+S+\frac{S^{2}}{K_{\text{i}}}}$ (1)

$v=\frac{v_{\text{m}}S}{K_{\text{m}}+S}$ (2)

where *v* (mmol N/(g MLSS·d)) represents the specific reaction rate; *v*_m_ (mmol N/(g MLSS·d)) is the maximum specific reaction rate; *S* (mM) is the substrate (NH_4_^+^-N) concentration; *K*_m_ (mM) is the apparent half saturation constant; *K*_i_ (mM) is the apparent inhibition constant. **Table S1. Conditions of batch tests.**

| Factor | Gradient | Other conditions |
| --- | --- | --- |
| pH | 3/4/5/6/7/8/9 | *Ⅽ*_inital NH₄⁺-N_=20 mg/L, *Ⅽ*_inital NO₂⁻-N_=0 mg/L,  T=25℃, SAL=0% |
| NH_4_^+^-N (mg/L) | 1/3/5/10/20/30/50/80/120/200/400 | pH=7.0±0.1, *Ⅽ*_inital NO₂⁻-N_=0 mg/L,  T=25℃, SAL=0% |
| NO_2_^-^-N (mg/L) | 10/20/30/50/80/100 | pH=6.0±0.1, *Ⅽ*_inital NH₄⁺-N_=20 mg/L,  T=25℃, SAL=0% |
| T (℃) | 15/20/25/30/35/40 | pH=7.0±0.1, *Ⅽ*_inital NH₄⁺-N_=20 mg/L,  *Ⅽ*_inital NO₂⁻-N_=0 mg/L, SAL=0% |
| SAL (%) | 0/0.25/0.5/1/2/3 | pH=7.0±0.1, *Ⅽ*_inital NH₄⁺-N_=20 mg/L,  *Ⅽ*_inital NO₂⁻-N_=0 mg/L, T=25℃ |

**Table S2. Trace element solution composition.**

| Reagent | Concentration (mg/L) |
| --- | --- |
| FeSO_4_·7H_2_O | 172 |
| ZnCl_2_ | 20 |
| MnCl_2_·4H_2_O | 47 |
| H_3_BO_3_ | 6 |
| CuSO_4_·5H_2_O | 3 |
| Na_2_MoO_4_·2H_2_O | 2 |
| NaCl | 584 |
| KCl | 746 |
| MgSO_4_·7H_2_O | 2465 |
| CaCl_2_·2H_2_O | 1470 |

**Table S3. Overview of substrate affinities of ammonia-oxidizing microorganisms reported in literatures.**

| Class | Culture | *K*_m_ (*μ*M NH_3_) | pH_M_ | pH_L_ | Reference |
| --- | --- | --- | --- | --- | --- |
| Comammox | **Comammox *Nitrospira* enrichment culture** | **0.5** | **7.0** | **/** | **This study** |
|  | *Nitrospira inopinata* (pure culture) | 0.063 | 7.5 | / | [2] |
|  | *Ca.* Nitrospira kreftii | 0.04 | 7.5 | / | [3] |
| AOB | **“*Ca.* Nitrosacidococcus. tergens” sp. RJ19 enrichment** | **0.147** | **4.7** | **2.5** | [4] |
|  | **“*Ca.* Nitrosoglobus. terrae” sp. TAO100** | **33.3** | **6.0** | **5** | [5] |
|  | *Nitrosomonas* *europea* (pure culture) | 36.47 | 7.6 | / | [6] |
|  | *Nitrosomonas* *mobilis* Ms1 (pure culture) | 1.54 | 8.0 | / | [7] |
|  | *Nitrosospira* *briensis* (pure culture) | 1.8-3 | 7.5 | / | [8] |
|  | *Nitrosococcus* *oceani* (pure culture) | 8.1 | 8.1 | / | [9] |
| AOA | ***Ca.* Nitrosotalea devanaterra** | **0.00069** | **5.3** | **4.0** | [10, 11] |
|  | ***Ca.* Nitrosotalea sinensis Nd2** | **0.00234** | **5.3** | / | [10] |
|  | *Ca.* *Nitrosotenuis uzomemsis* N4 (pure culture) | 0.54 | 7.5 | / |  |
|  | *Ca.* Nitrosocosmicus oleophilus MY3 | 9.23 | 7.0 | / |  |
|  | *Ca.* Nitrosopumilus adriaticus NF5 | 0.0045 | 7.3 | / |  |
|  | *Nitrosopumilus* *maritime* SCM1 (pure culture) | 0.0038 | 7.5 | / | [12] |
|  | *Candidatus* Nitrosoarchaeum koreensis MY1 | 0.0045 | 7.0 | / | [13] |
|  | *Candidatus* Nitrosoarchaeum koreensis AR enrichment | 0.069 | 8.2 | / | [14] |

pH_M_ is the pH corresponding to K_m_. pH_L_ is the lowest pH to support microbial growth.


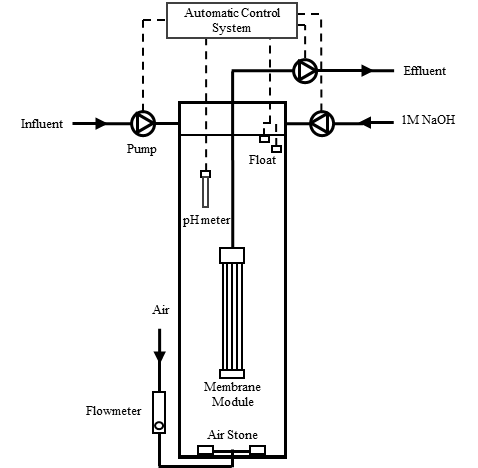


**Figure S1.** Diagram of the membrane bioreactor.

**Figure S2.** Nitrification performance of MBR. (A) Influent ammonium, effluent ammonium, nitrate, and nitrite concentrations; (B) Effluent pH value and calculated FA concentrations. The blue solid asterisks and blue hollow asterisks represent the sampling time for metagenomic and metatranscriptomic sequencing, respectively.

**
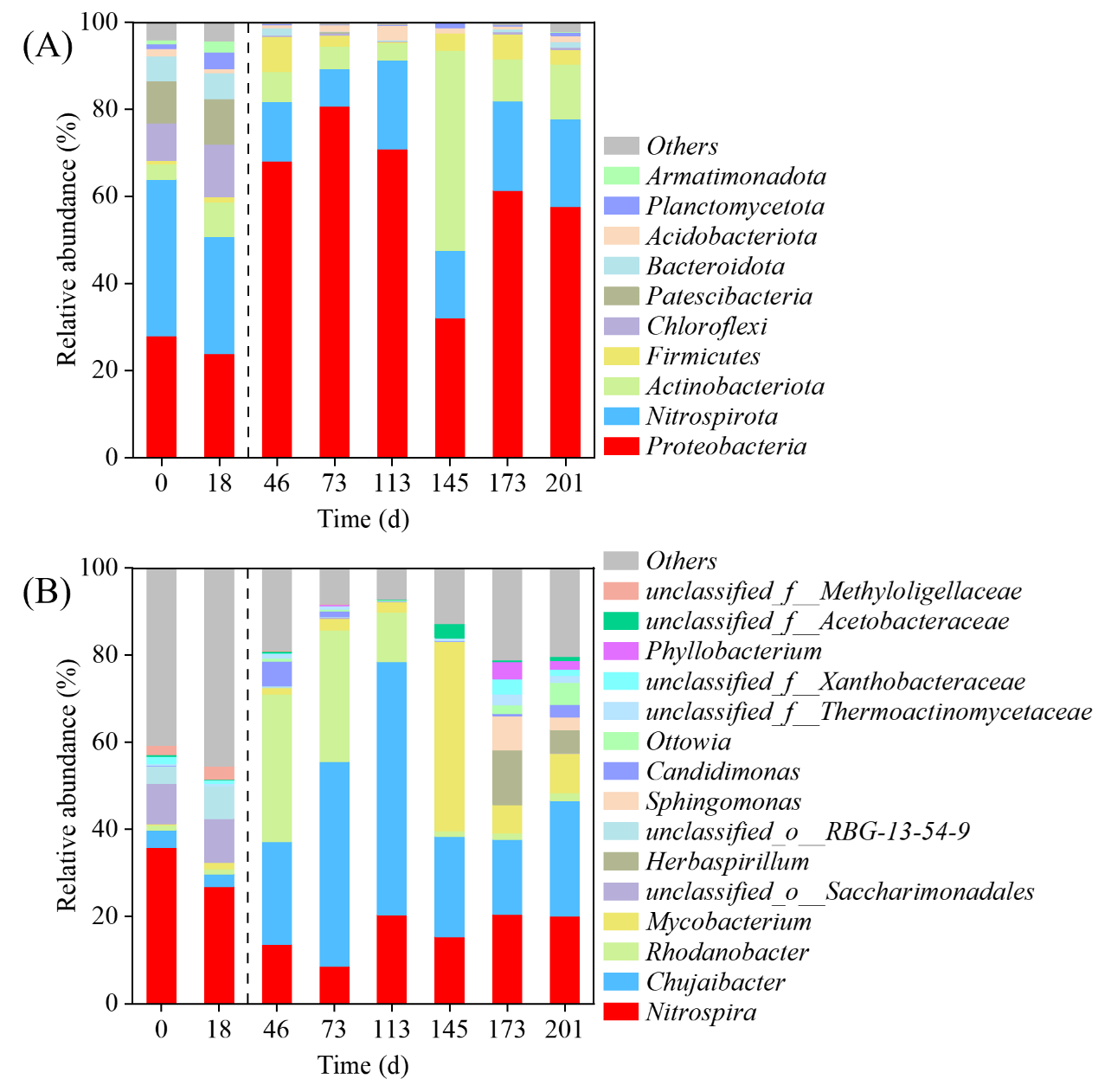
**

**Figure S3.** Variation in the top 10 phylum-level (A) and top 15 genus-level (B) amplicon sequence variants (ASVs) in the microbial community of the bioreactor during the whole culturing stage.


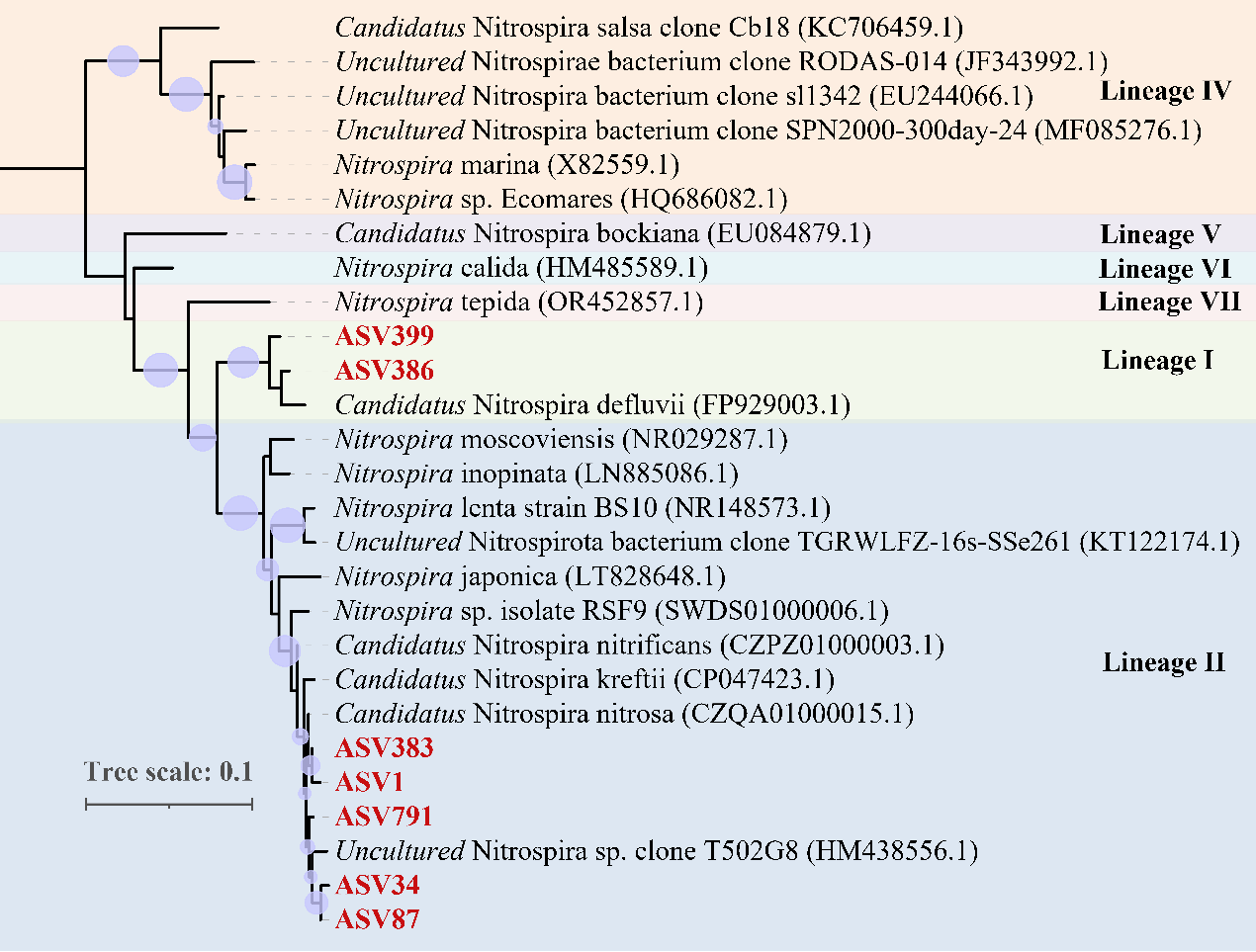


**Figure S4.** Phylogenetic analysis based on 16S rRNA gene amplicon sequencing. Maximum likelihood tree showing the phylogenetic affiliation of nitrifier 16S rRNA gene sequences obtained from the bioreactor. Circles on tree nodes denote the confidence of branching topology with 1000 replicates, and bootstrap values are indicated in the size of the purple circle.

**
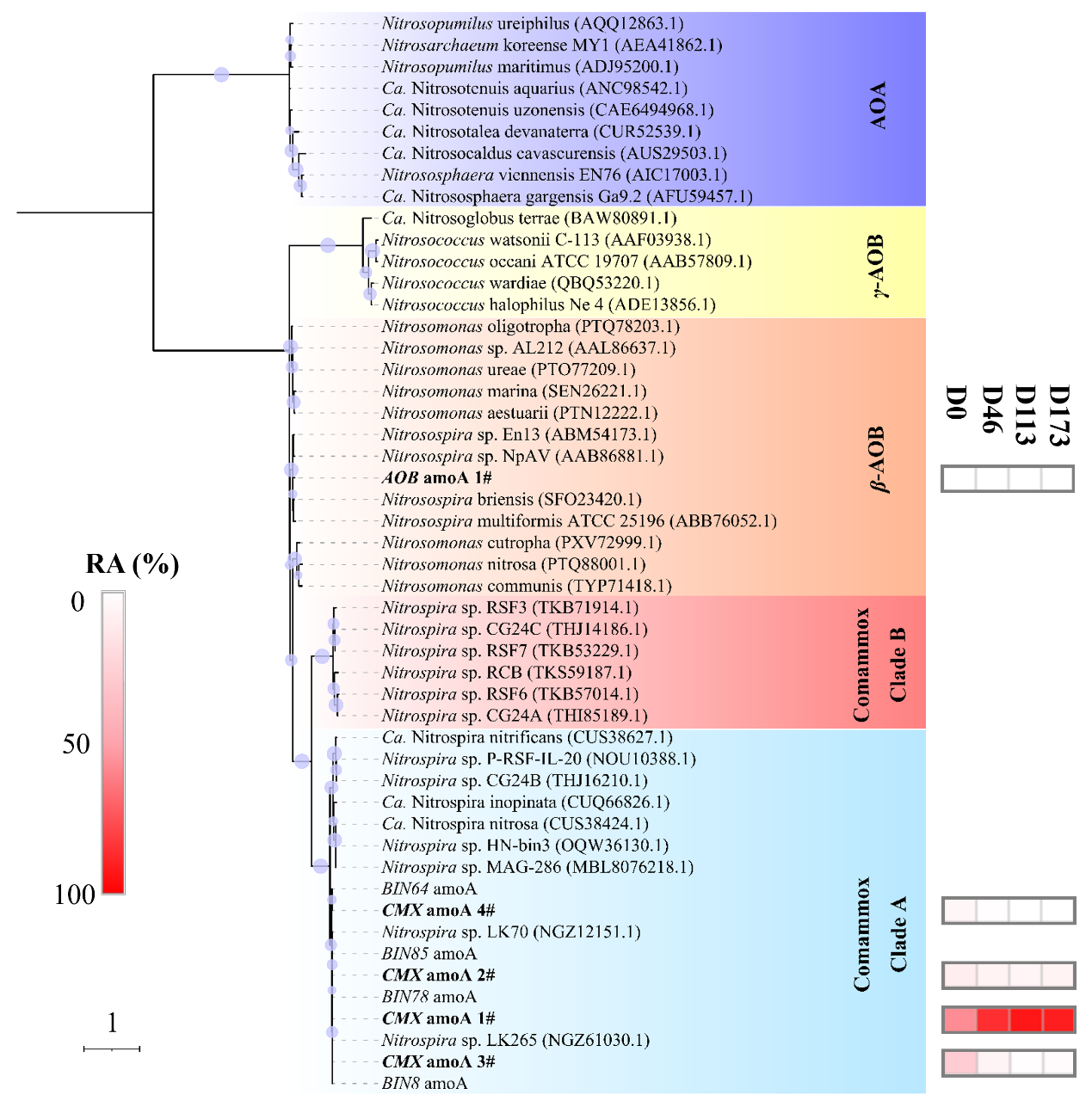
**

**Figure S5.** Phylogenetic tree of *amoA* gene sequences from comammox *Nitrospira*, AOB, and AOA, rooted from the AOA group. The heatmap show the relative abundance (RA) of each amoA gene recovered from metagenomic sequencing. Nodes with bootstrap values of ≥ 70% are marked with purple circles.


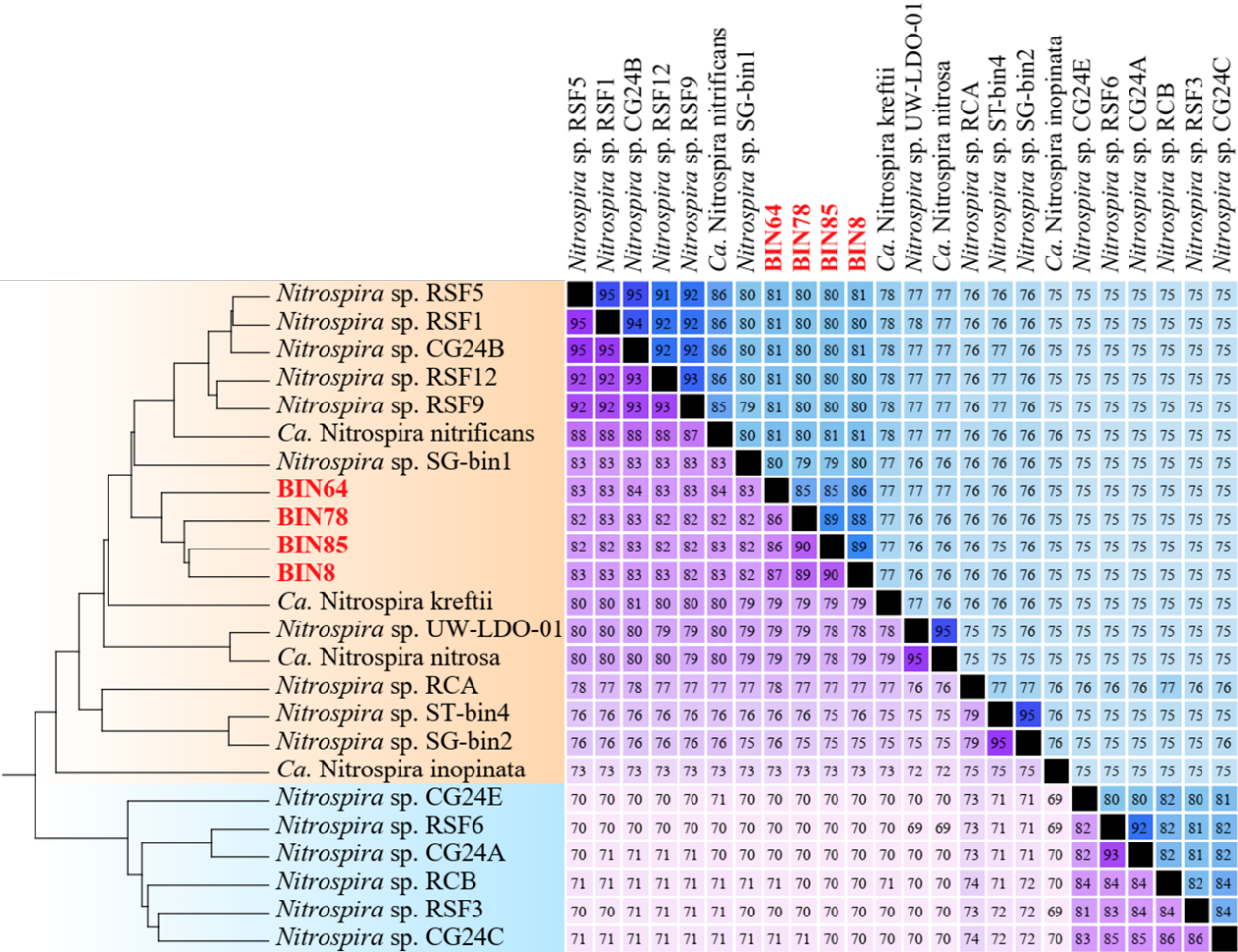


**Figure S6.** The average nucleotide identity (ANI) and amino acid identity (AAI) calculated for comammox *Nitrospira* clade A (orange) and B (blue) genomes. The sub-tree on the left was extracted from the phylogenetic tree in Figure 2. Heatmaps at the bottom right and at the top left give the average ANI and ANI values between all 28 comammox *Nitrospira* genomes, respectively. Genomes retrieved in this study are shown in red.


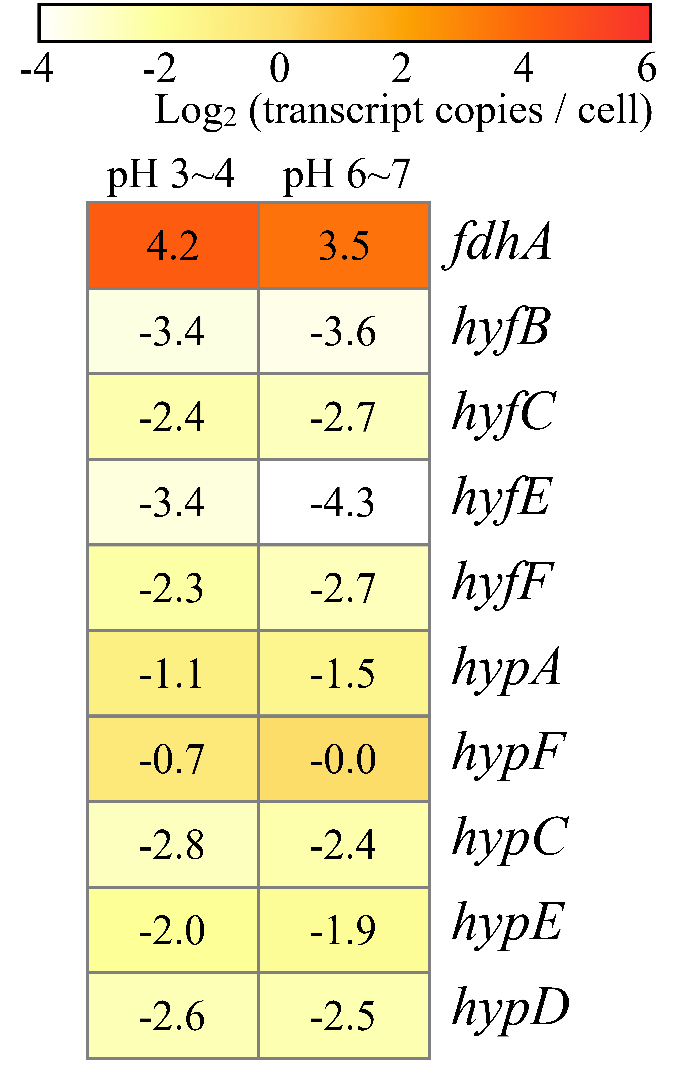


**Figure S7.** Differential gene expression profiles of *Ca.* Nitrospira aciditolerans involved in formic acid and hydrogen metabolism. The colors in heatmap show the differences in the mean transcript abundances per organism under acidic (pH 3~4, left) versus near-neutral (pH 6~7, right) conditions. Differences were computed using log2- transformed values.

**
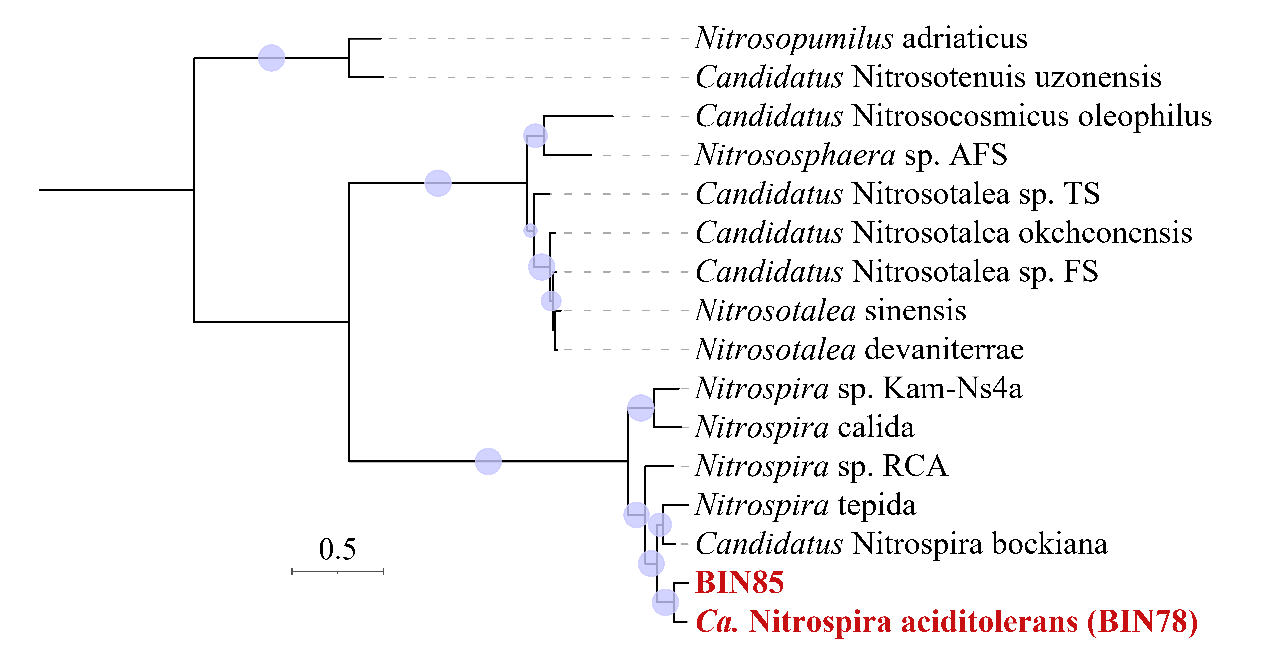
**

**Figure S8.** Maximum likelihood tree of the V-type ATPases based on the concatenation of the 6 subunits (A, B, D, E, F, and I) present in the “*Ca.* Nitrospira aciditolerans” genome and reference sequences. Bootstrap values (based on 1000 iterations) ≥ 70 are indicated by purple circles.

**
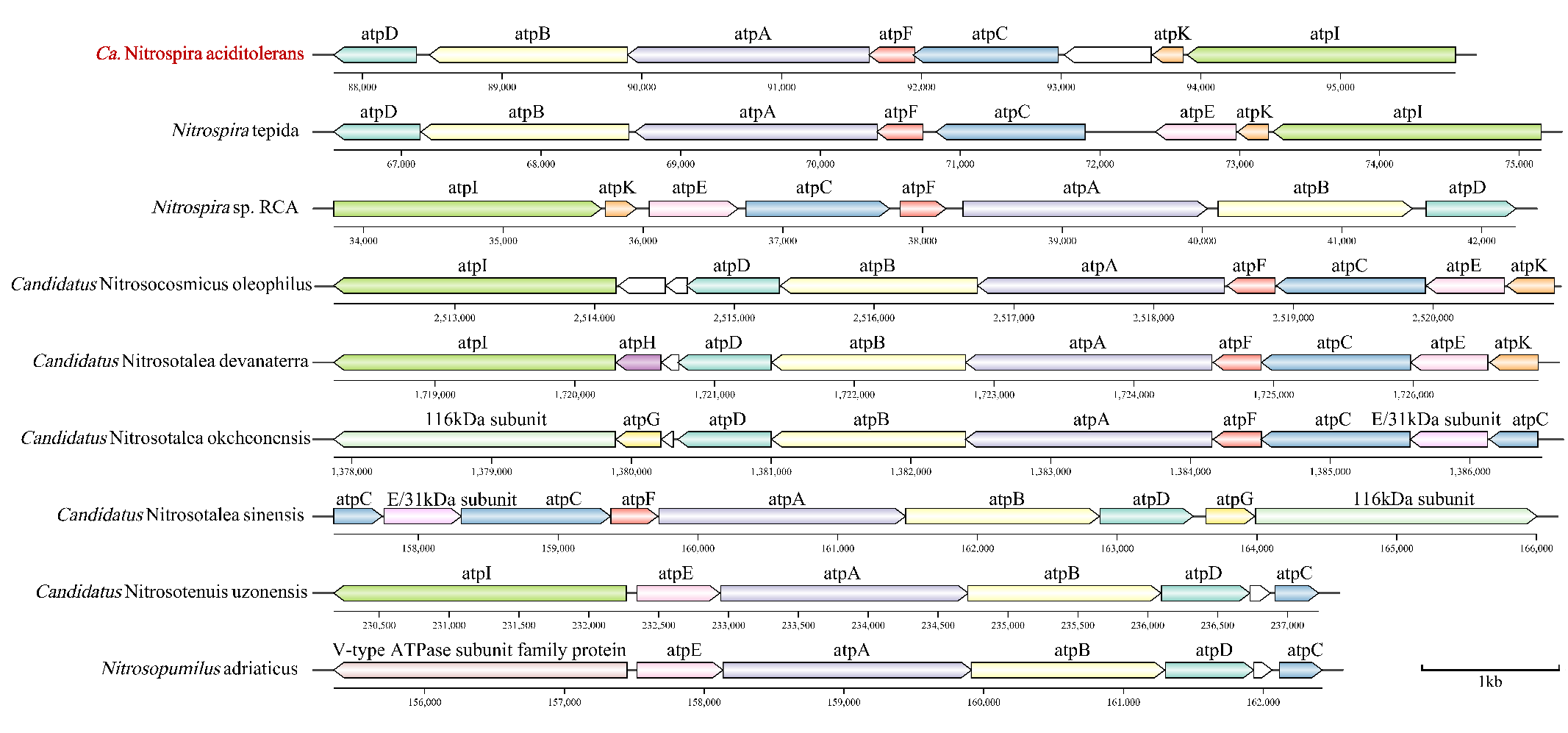
 Figure S9.** Schematic illustration of the genomic loci of the V-type ATPase with genes *atpA-I* of the “*Ca.* Nitrospira aciditolerans” genome and other AOA and *Nitrospira* genomes. Genes are drawn to scale

**
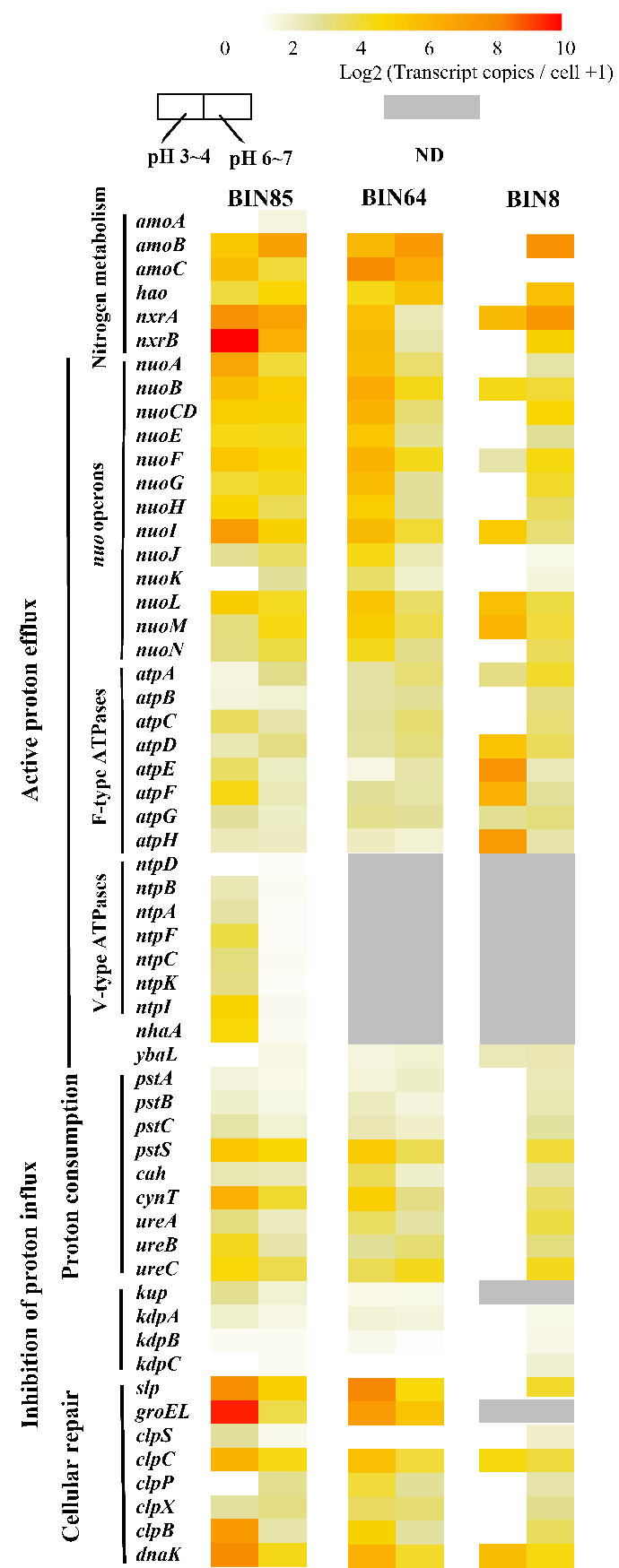
**

**Figure S10.** Differential gene expression profiles of three comammox *Nitrospira* MAGs involved in nitrogen metabolism and acid adaptation. The colors in heatmap show the differences in the mean transcript abundances per organism under acidic (pH 3~4, left) versus near-neutral (pH 6~7, right) conditions. Differences were computed using log2- transformed values.Gray boxes indicate undetectable gene expression.

**
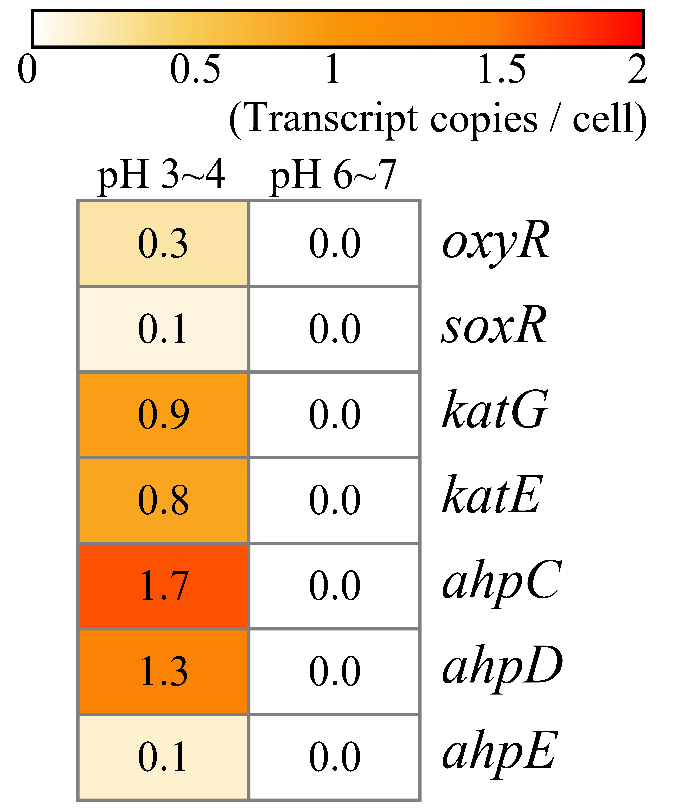
**

**Figure S11.** Differential gene expression profiles of *Mycobacterium* involved in oxidative stress. The colors in heatmap show the differences in the mean transcript abundances per organism under acidic (pH 3~4, left) versus near-neutral (pH 6~7, right) conditions.

**References**

1. Pan J, Li J, Zhang T *et al.* Complete ammonia oxidation (comammox) at pH 3–4 supports stable production of ammonium nitrate from urine. *Water Res* 2024;**257**:121686. <https://doi.org/https://doi.org/10.1016/j.watres.2024.121686>

2. Kits KD, Sedlacek CJ, Lebedeva EV *et al.* Kinetic analysis of a complete nitrifier reveals an oligotrophic lifestyle. *Nature* 2017;**549**:269-72. <https://doi.org/10.1038/nature23679>

3. Sakoula D, Koch H, Frank J *et al.* Enrichment and physiological characterization of a novel comammox Nitrospira indicates ammonium inhibition of complete nitrification. *ISME J* 2021;**15**:1010-24. <https://doi.org/10.1038/s41396-020-00827-4>

4. Picone N, Pol A, Mesman R *et al.* Ammonia oxidation at pH 2.5 by a new gammaproteobacterial ammonia-oxidizing bacterium. *ISME J* 2020;**15**:1150-64. <https://doi.org/10.1038/s41396-020-00840-7>

5. Hayatsu M, Tago K, Uchiyama I *et al.* An acid-tolerant ammonia-oxidizing γ-proteobacterium from soil. *ISME J* 2017;**11**:1130-41. <https://doi.org/10.1038/ismej.2016.191>

6. Kantartzi S, Vaiopoulou E. Kinetic characterization of nitrifying pure cultures in chemostate. *Global NEST Journal Copyright©* 2006;**8**:43-51.

7. Thandar SM, Ushiki N, Fujitani H *et al.* Ecophysiology and comparative genomics of Nitrosomonas mobilis Ms1 isolated from autotrophic nitrifying granules of wastewater treatment bioreactor. *Front Microbiol* 2016;**7**:1869. <https://doi.org/10.3389/fmicb.2016.01869>

8. Bollmann A, Schmidt I, Saunders AM *et al.* Influence of starvation on potential ammonia-oxidizing activity and amoA mRNA levels of Nitrosospira briensis. *Appl Environ Microbiol* 2005;**71**:1276-82. <https://doi.org/10.1128/aem.71.3.1276-1282.2005>

9. Ward BB. Kinetic studies on ammonia and methane oxidation by Nitrosococcus oceanus. *Arch Microbiol* 1987;**147**:126-33. <https://doi.org/10.1007/BF00415273>

10. Jung M-Y, Sedlacek CJ, Kits KD *et al.* Ammonia-oxidizing archaea possess a wide range of cellular ammonia affinities. *ISME J* 2022;**16**:272-83. <https://doi.org/10.1038/s41396-021-01064-z>

11. Lehtovirta-Morley LE, Stoecker K, Vilcinskas A *et al.* Cultivation of an obligate acidophilic ammonia oxidizer from a nitrifying acid soil. *Proc Natl Acad Sci USA* 2011;**108**:15892-97. <https://doi.org/doi:10.1073/pnas.1107196108>

12. Martens-Habbena W, Berube PM, Urakawa H *et al.* Ammonia oxidation kinetics determine niche separation of nitrifying archaea and bacteria. *Nature* 2009;**461**:976-9. <https://doi.org/10.1038/nature08465>

13. Jung M-Y, Park S-J, Min D *et al.* Enrichment and characterization of an autotrophic ammonia-oxidizing archaeon of mesophilic crenarchaeal group I.1a from an agricultural soil. *Appl Environ Microbiol* 2011;**77**:8635-47. <https://doi.org/10.1128/aem.05787-11>

14. Park BJ, Park SJ, Yoon DN *et al.* Cultivation of autotrophic ammonia-oxidizing archaea from marine sediments in coculture with sulfur-oxidizing bacteria. *Appl Environ Microbiol* 2010;**76**:7575-87. <https://doi.org/10.1128/aem.01478-10>
